# Administering human immunodeficiency virus post-exposure prophylaxis: challenges experienced by mothers in Lusaka, Zambia

**DOI:** 10.4102/sajhivmed.v22i1.1183

**Published:** 2021-01-27

**Authors:** Mildred Lusaka, Talitha Crowley

**Affiliations:** 1 Department of Nursing and Midwifery, Faculty of Medicine and Health Sciences, Stellenbosch University, Cape Town, South Africa

**Keywords:** mother-to-child transmission of HIV, post-exposure prophylaxis, prevention of mother-to-child-transmission of HIV, challenges

## Abstract

**Background:**

Mothers living with human immunodeficiency virus (HIV) should be guided to practise safe childbirth, provide appropriate infant feeding, return infants for repeat HIV testing and administer for the required period, protective antiretroviral (ARV) medication (post-exposure prophylaxis [PEP]) to their infants. Although several studies have explored challenges related to the prevention of mother-to-child transmission (PMTCT), no studies were found that focused specifically on the mother and PEP.

**Objectives:**

To explore and understand the challenges experienced by mothers in Lusaka, Zambia, whilst providing their children with PEP.

**Methods:**

This study utilised a qualitative methodology and a descriptive design. Fifteen semi-structured individual interviews were conducted with mothers who gave PEP to their infants. Study evaluation made use of Creswell’s six steps of data analysis.

**Results:**

Women experienced numerous challenges. Challenges of an individual and social nature included ‘negative’ emotions, misconceptions and a lack of understanding of PEP. Post-exposure prophylaxis was sometimes burdensome and partner involvement often limited. Cultural, religious practices and stigma deterred some women from continuing PEP. Healthcare challenges included time-consuming appointments and protracted waiting periods. Clinic organisation was often inefficient and complicated by stock-outs of essential medication such as nevirapine. Healthcare workers were at times stigmatising towards mothers living with HIV and their infants. The counselling support provided by the healthcare workers was felt to be inadequate in the face of the burden of PEP.

**Conclusion:**

Post-exposure prophylaxis as part of the PMTCT programme is key to eliminating mother-to-child transmission of HIV. Postnatal support for women administering PEP to their children can be enhanced through counselling that is person- and family-centred is culturally sensitive and offers differentiated services that include PEP, integrated mother-and-child healthcare and access to support groups.

## Introduction

Approximately 90% of new human immunodeficiency virus (HIV) infections of children and most acquired immunodeficiency syndrome (AIDS)-related paediatric deaths occur in Africa.^1,2^ In 2019, 150 000 children globally were ‘newly infected’ with HIV.^1^ Most acquire HIV infection vertically, that is, from their mothers. The UNAIDS target of eliminating mother-to-child transmission in Africa by 2030 is still a long way off.^2^ Numerous randomised clinical trials (RCTs) have confirmed the efficacy of antiretroviral (ARV) drugs both as treatment and as prevention of HIV.^3^ The widespread and consistent use of ARVs in prevention, antiretroviral treatment (ART) and infant–mother post-exposure prophylaxis (PEP) provides Africa with its most accessible means to end HIV-related infant morbidity and mortality.^1,4^

Most African countries have implemented prevention of mother-to-child transmission (PMTCT) programmes since 1999.^5^ In 2016, Zambia adopted the Option B+ plan that provides all pregnant and breastfeeding women (mothers) living with HIV (WLWH) lifelong ART, regardless of their CD4 count or clinical stage.^6^ These WLWH protect their infants by taking maternal ART throughout pregnancy and breastfeeding, and by giving their HIV-exposed infant ARVs immediately after birth and until 28 days following the end of breastfeeding. Maternal viral load (VL) monitoring measures the protective efficacy of the programme.^7^ The use of ARVs in this way is infant- PEP and is an important component of the PMTCT programme in Zambia.

Prevention of mother-to-child transmission programmes prevent infant HIV infections and deaths.^2^ Nevertheless, women participating in these programmes in Malawi, Mozambique and South Africa report individual and socio-economic challenges, such as a lack of knowledge about infant ART, transportation costs, inadequate spousal or family support and a fear to disclose because of HIV/AIDS-related stigma and discrimination.^8,9,10,11,12,13^

The poor quality of postnatal services in Africa’s generally weak healthcare systems and the challenges faced by individual women and their socio-economic circumstances compromise PMTCT retention rates.^2,14^ Few PMTCT programmes provide support, treatment and ongoing care for the infant after discharge.^14^ In Zambia and India, reported inadequacies include a lack of information and professional support, a lack of HIV-testing kits and shortages of ARVs and infant nevirapine (NVP).^15,16^ Women on ART during pregnancy experience a unique postpartum transition from receiving care for themselves and the baby simultaneously, whilst the infant is, *in utero*, to receiving separate infant and maternal care after delivery.^11^ In addition, the infant’s medication (PEP) must be given as medically prescribed. There is therefore a particular need to support WLWH whilst in the postnatal and breastfeeding period.^2,17^

Although several studies have explored the experiences of women engaged in PMTCT with some focusing on the postnatal period, none has focused specifically on the challenges that mothers experience in providing PEP to their infants after birth. The administration of PEP to infants is a key pillar of the PMTCT programme. This has made it imperative to explore the challenges that mothers experience when administering PEP to their infants.

## Methods

### Study design

A qualitative approach with an explorative-descriptive design was used. Administering PEP to a baby by a mother living with HIV is a complex and multi-faceted phenomenon. A qualitative design was selected to gain a detailed in-depth understanding of these challenges.

### Setting

The study was conducted at the University Teaching Hospital (UTH) in Lusaka, the capital city of Zambia. The UTH is the principal training centre for healthcare workers of different categories and the main referral hospital of the country.

### Population and sampling

The target population was adult mothers living with HIV who had been caring for an infant since birth and had experience of administering PEP within the 3 months prior to data collection. A purposive sample of 15 women who attended their 6 weeks postnatal visit was drawn to participate in the study. Purposive sampling aimed to include women from various settings around Lusaka, different language and socio-economic backgrounds, those with prior experience in providing PEP and those without, to ensure maximum variability of experiences.^18^ The study focused on adult mothers because there may be specific challenges unique to adolescent mothers. The hospital staff first obtained permission from possible participants before referring them to the researcher. Data saturation was reached when the 15th interview yielded no new information.

### Data collection

In-depth interviews were undertaken in August and September 2016 using a semi-structured interview guide. The first author, who was not personally known to the participants or involved in their care, conducted one-on-one interviews at a place convenient for the participants. She was trained in conducting individual interviews, which was done in the women’s home language, Nyanja or Bemba; some participants spoke English in-between the conversation. Interviews took place in a separate room within the UTH building but in another department to maintain privacy. Open-ended questions were asked in an informal, conversational manner, in order to allow the participants to talk freely about their experiences. Interviews lasted between 35 and 45 min, were audio-recorded and field notes were taken. As member-checking was done in the interviews by means of summarising and clarifying information, and data saturation was achieved, no follow-up interviews were required.

### Data analysis

Data analysis was performed simultaneously throughout data collection. The first author listened to the recordings and transcribed the participants’ recorded interviews verbatim. This was then translated into English. Data were analysed manually, organised and interpreted using Creswell’s six-step technique of data analysis for qualitative research.^19^ All information collected from the participants through interviews were analysed by both authors by way of gathering and generating the collected data into themes. Discrepancies were resolved through consensus.

Trustworthiness was maintained through adhering to the principles of credibility, transferability, conformability and dependability.^19^ Credibility was ensured through selection of those participants who met the inclusion criteria and by following the interview guide. Assurance regarding the truth of data collected was established through the techniques of peer debriefing and member checks. Transferability was pursued during data collection until data saturation occurred and a description was provided of the study setting. Conformability was established through the recorded data that substantiated the findings and that agreed that the interpretation was a genuine reflection of the participants’ experiences. Dependability is supported by a step-by-step ‘audit trail’.

### Ethical consideration

Ethical approval to conduct the research study was obtained from the Health Research Ethics Committee (HREC) of the Faculty of Medicine and Health Sciences, Stellenbosch University (SU), South Africa (SA), Reference number S16/04/062. In addition, approval was obtained from the Permanent Secretary, Ministry of Health in Zambia, the Institution and Head of Department of the University Teaching Hospital in Lusaka, Zambia. Further ethical approval was obtained from the Biomedical Health Research Ethics Committee (BHREC), University of Zambia (UNZA), Reference number 001-07-16. Written informed consent was obtained from all participants. Confidentiality was maintained throughout the study to protect the HIV status of participants by using identification numbers in lieu of personal identification. These identification numbers were also used when transcribing the interviews.

## Results

### Participant characteristics

Fifteen participants were enrolled in the study. The participants were mothers from peri-urban and urban areas around Lusaka city who were administering ART to their infants at the time of the study. Nine participants were married (9/15 = 60%), two were divorced, one was separated from her partner and three were single mothers. The participants’ age ranged from 20 to 42 and they had two to four children. Three participants were employed and 12 were unemployed (see Table 1).

**TABLE 1:** Participant characteristics.

Variable	Values
**Marital status**	
Married	9
Single	6
Mean age	30.7
Average number of children	2.9
**Employment**	
Employed	3
Unemployed	12

### Themes

Seven themes with several sub-themes were identified as depicted in Table 2.

**TABLE 2:** Themes and sub-themes.

Themes	Sub-themes
Emotional responses	Apprehension Self-reproach
Knowledge and understanding	Sources of information Misconceptions about PEP and breastfeeding
PEP administration practices	Hiding Burden
Healthcare appointments	Time consuming Behaviour of healthcare workers
Partner involvement	Support Abandonment
Cultural and religious influences	Family roles and support Beliefs and customs Religious beliefs (faith healing)
Stigma and discrimination	Disclosure Healthcare settings

*Source:* Lusaka, Master’s thesis Stellenbosch University

PEP, post-exposure prophylaxis.

### Emotional responses

Participants expressed several emotional responses towards living with HIV, the possibility of transmitting the virus to the infant, providing PEP and breastfeeding. For some participants, it brought apprehension, fear, worry and a sense of guilt realising that it would be their fault if their child became HIV-positive. Yet, they tended to manage these emotions alone:

‘I am fearful of losing my baby even if I am giving nevirapine.’ (Participant 2, 31 years old, married)

Some participants were worried about the side effects of NVP, the drug given to the infant for PEP and the potential harm that it could cause to their infants. The awareness that high level of adherence was needed in order to prevent HIV transmission made participants worry when their babies did not swallow all the medication:

‘The baby doesn’t swallow all the medication … it is scary to keep waiting for the results when you know that the baby never swallows all the medication at times. I don’t want to be told that the baby is positive.’ (Participant 1, 20 years old, single)

Some participants expressed feelings of guilt and self-blame as they felt responsible for the suffering of the infant. Feelings of guilt and self-reproach also emanated from experiences of not adhering to the advice of healthcare workers during previous pregnancies:

‘Help was offered to me, but I chose to ignore it. I did not want others to know, it was so foolish of me. I should have composed myself enough to do the right thing … I lost my three babies probably because of being HIV-positive or because of not following what the nurses told us from the antenatal clinics.’ (Participant 6, 38 years old, married)

### Knowledge and understanding

Some of the participants were sceptical about the efficacy of PEP. The reasons for their doubt appeared to be a lack of knowledge and understanding, as well misconceptions about the need for providing PEP and its benefits. Some participants were misinformed about breastfeeding and they expressed ignorance of safe breastfeeding practices. Many of these misconceptions were because of conflicting information received from different sources. Participants received information from their church pastors, mother-in-law’s, family members, friends and healthcare workers:

‘I hear from friends that the medication results in complications.’ (Participant 9, 22 years old, single)

Some participants had inadequate information because of fear of asking healthcare workers if they do not understand:

‘I was scared of being scolded at by health professionals. The instructions are scribbled on the bottle. At times the amount changes and is not visible enough to read.’ (Participant 11, 27 years old, single)

The perceptions of some participants were that PEP was unnecessary because their infants were healthy. One participant believed that as her baby looked healthy, there was no need to give the drug:

‘The baby looks healthy, why give the nevirapine?’. (Participant 2, 31 years old, married)

In some instances, the administration of NVP was believed to be associated with cancer and diarrhoea. These misconceptions influenced the willingness of the mothers to give NVP to their babies:

‘I am told by friends that these drugs cause cancer of the liver, these drugs are so strong, and ward 6 has a number of children with cancer.’ (Participant 9, 22 years old, single)

### Post-exposure prophylaxis administration practices

Participants who had disclosed their HIV status were less likely to report having problems administering NVP.

For some, giving NVP to their infants was a burden because the act of giving medication was a constant reminder of HIV infection. Many of the participants admitted that they chose to hide the administration of PEP from their family members, friends and partners. This was done by removing the medicine label from the bottle or by waiting until they were alone:

‘At first, I would remove the label from the bottle and I would give the medicine to the baby when I was alone.’ (Participant 6, 38 years old, married)

Participants found it difficult to adhere to treatment and postnatal appointments when they continued hiding the administration of NVP to their infants from family and friends. The constant thought of having to give NVP was expressed as a burden and remembering to give the medication on a daily basis or making arrangements when the mother was not available to give the medication was a concern:

‘I had challenges telling her the truth. There are times when I would want my stepchild to give the medication to the baby. And I would just instruct her and not tell her what it was. She is a grown-up girl. One day I told her that it was medicine for diarrhoea, please give the baby when she wakes up. I was the only one who could give the medication.’ (Participant 10, 29 years old, married)

### Healthcare appointments

Some participants mentioned being unhappy with hospital appointments and complained that time was wasted whilst waiting for services. Inefficient organisation and the long waiting times at the facilities and when obtaining HIV test results were barriers to accessing postnatal care:

‘There were long queues – baby still too small, those that were to receive vaccines and results were put in the same line, takes time to have one’s turn. Babies who were successfully immunised and whose HIV tests were done for the first time still go back to line up for supply of NVP.’ (Participant 12, 27 years old, single)

Working mothers found it difficult to get permission from work to attend hospital appointments, especially because the healthcare appointments for herself and her baby were separate:

‘It was not easy to get permission from work. I used to get permission for myself and now I have to get permission for me and the baby … Not easy to get permission from work to go for hospital.’ (Participant 5, 32 years old, married)

Drug stock-outs contributed further to the time being spent on healthcare appointments:

‘Only medicine for 4 days was given at times and I was required to get permission twice within a week to get back to the hospital for more medicines.’ (Participant 3, 26 years old, married)

Participants perceived some healthcare workers as uncaring, non-sympathetic and unapproachable. Stigmatising behaviour by healthcare workers was a challenge mentioned by some participants:

‘Nurses would give each other signs, a certain way of communication among themselves referring to HIV-positive mothers.’ (Participant 3, 26 years old, married)

### Partner involvement

Some participants feared abandonment if they disclosed their HIV status to their partners, while others mentioned that their partners refused to test for HIV and did not support them emotionally:

‘I did not know how to tell my husband. As such I was in constant fear because I could not guess how he was going to react. I kept quiet and pretended that everything was fine.’ (Participant 7, 29 years old, married)

‘It is really unfortunate, that my husband was in a position not to listen … I think he knew that he was positive, that was why he was so defensive, and you know he knew that he was positive even before we got married.’ (Participant 6, 38 years old, married)

### Cultural and religious influences

In the Zambian context, the family plays a pivotal role in the culture of child nurturing. The influence of the extended family, including grandmothers and the mother-in-law, played a significant role in the decisions made by participants to attend healthcare appointments for PEP. Moving around in public with a baby below the age of 3 months is considered unacceptable in some Zambian cultures and motivating to attend appointments without disclosing their HIV status was a challenge for women:

‘But my mother-in-law believes in staying indoors until the baby is 3 months old.’ (Participant 6, 38 years old, married)

‘I didn’t go for the 1-week appointment. I was told the baby was too young … my mother-in-law escorted me when the baby was 6 weeks. She told me that the baby was too young to be moving around with her in public.’ (Participant 11, 20 years old, single)

Participants appeared to have different personal beliefs about the administration of NVP and the impact that it would have on their infants. Some indicated that they believed in traditional medicine or spiritual healing.

In some instances, they expressed fear and mistrust of PEP, which was also negatively associated with demons.

Participants proved that they were not demon-possessed by seeking spiritual intervention. These negative beliefs led some participants not to administer NVP to their infants whilst they were seeking spiritual intervention:

‘We used to go for overnight prayers and believed that the anointing water and oil were going to cure us.’ (Participant 3, 26 years old, married)

### Stigma and discrimination

Stigma and discrimination were identified as a continuous challenge. It was very obvious that stigma and discrimination were widespread in families and communities. This forced women to avoid practices that revealed their HIV status. Much of the stigma was associated with misconceptions about HIV and the administration of NVP to the infants. Stigma and discrimination led participants to make decisions to either continue or to discontinue treatment.

A lack of privacy and confidentiality in healthcare settings was a concern. Some participants felt uncomfortable because they were told about NVP administration and HIV results within the hearing distance of others:

‘At the clinics, you are told to sit in a waiting space before being given the results. When you are many, a counsellor would come out and call out names, giving the papers carrying the negative results and after that she tells them to go. An announcement will be made for those remaining not to leave but to remain seated until results were given.’ (Participant 10, 29 years old, married)

## Discussion

Several individual, social and healthcare system challenges were identified that were related to the administration of PEP to infants born to WLWH. Individual challenges included emotional responses such as worry and fear, a lack of knowledge and understanding as well as misconceptions regarding PEP. Some reported that they did not know how to read the instructions provided. Others felt that instructions were unclear and that specific issues such as drug administration times and what to do in case of vomiting or spitting were unclear. Information about PEP administration was communicated through group counselling as well as pre- and post-test counselling. Yet, time constraints resulted in the information being given hastily and with little opportunity for questions and clarification. Similar anxiety about the infant’s HIV diagnosis has been identified in a South African study on PMTCT.^11^ Reports from Malawi, Ethiopia, Tanzania and Ghana also confirm that mothers lack knowledge of infant ART, feeding and how to care for their infants.^8,20,21,22,23,24^

Social challenges mainly related to cultural and religious influences, limited partner involvement, a lack of disclosure and support. Mothers in the current study were obliged to follow the advice of grandmothers, mothers and mothers-in-law.^20^ Various religious practices and the fear of stigma influenced the administration of PEP: all reported in other studies.^8,21,24^ Additional studies from sub-Saharan Africa provide a wide socio-economic backdrop of poverty, migration, transport costs and the failure of families to provide adequate support to ensure the effectiveness of infant PEP.^9,24,25^ The organisation of healthcare services caused inefficiencies and hindered the clarification of essential information. Participants experienced stigma, a lack of confidentiality and uncaring behaviour from healthcare staff. Indeed, the healthcare system itself was often a challenge to overcome rather than a supportive asset to these young mothers.^24,26,27^

These findings indicate the need for person or family-centred counselling that focuses on the evaluation of and solution to individual barriers and enablers of PEP. Counselling should also be culturally sensitive and assist women to incorporate their cultural and spiritual beliefs in a meaningful way into the care of their infants.

Family-counselling interventions and peer support groups could be a solution and facilitate family, peer and partner support.^28^ Although facility-based, mother-support groups in Zimbabwe failed to improve the retention in care of HIV-exposed infants at 12 months, it may still have a role in certain contexts and amongst high-risk groups.^28^ Integration of PEP into routine child healthcare visits or the combining of mother-and-child care may reduce the burden of multiple appointments. Providing differentiated care such as fast-tracking women, who need PEP refills only or providing community PEP refills, should be considered. A recent report suggests that the provision of correct education, family and partner involvement, addressing health systems gaps, linking the clinic with the community and the provision of income generation support is key to promoting adherence within PMTCT programmes.^24^

More qualitative and quantitative research is needed to explore PEP-related behaviours and to understand the factors that influence PEP administration over longer periods of time. This may be required during high-risk exposures.

## Strengths and limitations of the study

To the best of our knowledge, this is the only qualitative study to focus specifically on the experiences of WLWH whilst providing PEP to their infants. The literature focuses on factors influencing maternal ART adherence and less so on PEP administration, which is an equally important component of the PMTCT programme. There are several study limitations: the study was conducted at only one hospital and clinic, the UTH, and included only mothers who attended postnatal visits, which may limit transferability to other settings. At the time of the study, there was limited availability to published literature on the challenges of mothers administering PEP to their infants.

## Conclusion

The administration of PEP as part of the PMTCT programme is key to ensuring the goal of zero mother-to-child transmission. The findings indicate that women who administer PEP to their infants have to overcome a variety of individual, social and healthcare system challenges. Postnatal support for these women can be enhanced through person or family-centred, culturally sensitive counselling, providing differentiated services for PEP, integrated mother-and-child healthcare and access to support groups.
